# Supplementary material for: Evaluation of the Nutritional Status of Gaucher Disease Type I Patients under Enzyme Replacement Treatment
Source: Nutrients. 2022 Aug 3;14(15):3180. doi: 10.3390/nu14153180 (PMC9370155; doi:10.3390/nu14153180)
Supplement: Supplementary file 1 [file nutrients-14-03180-s001.zip › Table S1 molecular analysis GD1 patients.pdf]

Table S1: Molecular analysis of the patients with Gaucher disease

| Patient ID number | GBA gene mutation Allele 1 | GBA gene mutation Allele 2 |
|---|---|---|
| GD1 | N370S | L444P |
| GD2 | N370S | L444P |
| GD3 | N370S | W393R |
| GD4 | N370S | W393R |
| GD5 | N370S | L444P |
| GD6 | N370S | N370S |
| GD7 | R170C | 589-12C>G |
| GD8 | R170C | 589-12C>G |
| GD9 | N370S | AH006907.2:g.1942_7319con100042_13806 |
| GD10 | N370S | M85V |
| GD11 | R170C | 589-12C>G |
| GD12 | R353G | R353G |
| GD13 | R353G | R353G |
| GD14 | N370S | N188K |
| GD15 | W312C | W312C |
| GD16 | T43I | T43I |
| GD17 | T43I | T43I |
| GD18 | L444P | S364T |
| GD19 | L444P | S364T |
| GD20 | N370S | L444P |
| GD21 | N370S | L444P |
| GD22 | N370S | L444P |
| GD23 | N370S | L444P |
| GD24 | L444P | L444P |
| GD25 | N370S | L354X |
| GD26 | N370S | L354X |

GBA= glucocerebrosidase
