# Supplementary material for: Evaluation of the Nutritional Status of Gaucher Disease Type I Patients under Enzyme Replacement Treatment
Source: Nutrients. 2022 Aug 3;14(15):3180. doi: 10.3390/nu14153180 (PMC9370155; doi:10.3390/nu14153180)
Supplement: Supplementary file 1 [file nutrients-14-03180-s001.zip › Table S2 Manuscript Gaucher REE.pdf]

Table S2: Biochemical characteristics of the study population of patients with type I Gaucher disease.

	TOTAL n=26	MEN n= 11	WOMEN n=15	*p*
	**mean ± SD (min-max)**	**mean ± SD (min-max)**	**mean ± SD (min-max)**	
WBC (**10³/UL*)	7.1±3.3 (2.9-19.4)	7.7 ± 4.2 (3.6– 19.4)	6.7 ± 2.6 (2.9 – 11.4)	*0.467*
RBC *(*10⁶/UL)*	4.9±0.6 (3.2-6.2)	5.2 ± 0.4 (4.6 -6.2)	4.6 ± 0.6 (3.2 -5.9)	*0.009*
Haemoglobin (*g/dl*)	14.5±1.6 (10.6 –17.9)	14.9 ± 2.0 (10.6 – 17.9)	14.3 ± 1.2 (12.6 – 16.6)	*0.336*
Platelet (**10³/UL*)	190 ± 51 (80-297)	197 ± 48 (131 – 297)	186 ± 54 (80- 280)	*0.575*
Lymphocites *(*10³/UL)*	2.2±2.2 (0.9-13.2)	2.9 ± 3.5 (0.9 – 13.2)	1.8 ± 0.5 (0.9 –2.8)	*0.252*
Total Protein (*g/dl)*	7.4±0.6 (6.2-9.0)	7.4 ± 0.6 (6.3-9.0)	7.3 ± 0.7 (6.2 -9.0)	*0.642*
Alb (*g/dl)*	4.5±0.3 (4.0-5.3)	4.6 ± 0.3 (4.3 -5.3)	4.5 ± 0.3 (4.0 -5.1)	*0.200*
Tot-C (*mg/dl)*	169±29 (93-219)	172 ± 25 (137-219)	167 ± 32 (93-216)	*0.698*
TG (*mg/dl)*	96±55 (39-328)	93 ± 30 (46-141)	99 ± 68 (39-328)	*0.797*
LDL-c (*mg/dl)*	114±30 (51-178)	123 ± 28 (92 -178)	106 ± 31 (51 -150)	*0.176*
HDL-c (*mg/dl)*	48±11 (32-71)	43 ± 7 (33-56)	52 ± 11 (32 -71)	*0.021*
AST(*U/l)*	120±6 (11-38)	24 ± 7 (15- 38)	17 ± 4 (11- 25)	*0.002*
ALT *(U/l)*	22±13 (10-63)	31 ± 15 (15- 63)	15 ± 5 (10 – 31)	*0.001*
Glucose (*mg/dl)*	98±22 (62-168)	104 ± 24 (78- 168)	94 ± 21 (62- 140)	*0.274*
Insulin (*µU/mL*)	10.0±5.0 (1.0 - 23.6)	11.1 ± 3.6 (5.0-16.9)	9.4 ± 5.7 (1.0- 23.6)	*0.428*
Vit B12 *(pg/ml)*	374±101 (177 -547)	373 ± 88 (275 -538)	374 ± 113 (177 -547)	*0.981*
Folate *(ng/ml)*	6.0±3.3 (1.9-15.3)	5.5 ± 2.9 (2.4 -10.8)	6.4 ± 3.7 (1.9 -15.3)	*0.554*
CT Activity *(nmol/ml/h)*	409±620 (23-3081)	254±213 (23-716)	516±779 (66-3081)	*0.094*
Lyso-GB1 (ng/ml))	51.2± 34.9 (14.1-137.3)	46.6±31.6 (15.5-111.6)	54.2±37.6 (14.1-137.3)	*0.600*

Mean±SD; WBC: white blood cells; RBC: red blood cells; Alb , Albumine; Tot-C,total cholesterol; TG, triglycerides; LDL-c, low-density lipoprotein cholesterol; HDL-c, high-density lipoprotein cholesterol; AST aspartate aminotransferase ; ALT , alanine aminotransferase; CT Chitotriosidase; Lyso-GB1: glucosylsphingosine.
